# Mucosal immune responses in COVID19 - a living review

**DOI:** 10.1093/oxfimm/iqab002

**Published:** 2021-01-25

**Authors:** Claire F Pearson, Rebecca Jeffery, The Oxford-Cardiff COVID-19 Literature Consortium, David J Ahern, Hannah Almuttaqi, Dominic S Alonzi, Aljawharah Alrubayyi, Ghada Alsaleh, Valentina M T Bart, Vicky Batchelor, Rebecca Bayliss, Dorothée L Berthold, Jelena S Bezbradica, Tehmina Bharuchq, Helene Borrmann, Mariana Borsa, Rowie Borst, Juliane Brun, Stephanie Burnell, Lorenzo Capitani, Athena Cavounidis, Lucy Chapman, Anne Chauveau, Liliana Cifuentes, Amy Susan Codd, Ewoud Bernardus Compeer, Clarissa Coveney, Amy Cross, Sara Danielli, Luke C Davies, Calliope A Dendrou, Sandra Dimonte, Ruban Rex Peter Durairaj, Lynn B Dustin, Arthur Dyer, Ceri Fielding, Fabian Fischer, Awen Gallimore, Sarah Galloway, Anís Gammage, Ester Gea-Mallorquí, Andrew Godkin, Stephanie Jean Hanna, Cornelia Heuberger, Sarah Hulin-Curtis, Fadi Issa, Emma Jones, Ruth Jones, Kristin Ladell, Sarah N Lauder, Kate Liddiard, Petros Ligoxygakis, Fangfang Lu, Bruce MacLachlan, Shayda Maleki-Toyserkani, Elizabeth H Mann, Anna M Marzeda, Reginald James Matthews, Julie M Mazet, Anita Milicic, Emma Mitchell, Owen Moon, Van Dien Nguyen, Miriam O'Hanlon, Clara Eléonore Pavillet, Dimitra Peppa, Ana Pires, Eleanor Pring, Max Quastel, Sophie Reed, Jan Rehwinkel, Niamh Richmond, Felix Clemens Richter, Alice J B Robinson, Patrícia R S Rodrigues, Pragati Sabberwal, Arvind Sami, Raphael Sanches Peres, Quentin Sattentau, Barbora Schonfeldova, David Oliver Scourfield, Tharini A Selvakumar, Freya R Shepherd, Cariad Shorten, Anna Katharina Simon, Adrian L Smith, Alicia Teijeira Crespo, Michael Tellier, Emily Thornton, Lion F K Uhl, Erinke van Grinsven, Angus K T Wann, Richard Williams, Joseph D Wilson, Dingxi Zhou, Zihan Zhu, Emily E Thornton

**Affiliations:** Kennedy Institute of Rheumatology, NDORMS, University of Oxford, Roosevelt Drive, Oxford OX3 7FY, UK; Kennedy Institute of Rheumatology, NDORMS, University of Oxford, Roosevelt Drive, Oxford OX3 7FY, UK; Kennedy Institute of Rheumatology, NDORMS, University of Oxford, Roosevelt Drive, Oxford OX3 7FY, UK; MRC Human Immunology Unit, Weatherall Institute of Molecular Medicine, Nuffield Department of Medicine, University of Oxford, John Radcliffe Hospital, Headley Way, Oxford OX3 9DS, UK

**Keywords:** COVID-19, mucosal immunity, gut, lung, microbiome

## Abstract

COVID-19 was initially characterized as a disease primarily of the lungs, but it is becoming increasingly clear that the SARS-CoV2 virus is able to infect many organs and cause a broad pathological response. The primary infection site is likely to be a mucosal surface, mainly the lungs or the intestine, where epithelial cells can be infected with virus. Although it is clear that virus within the lungs can cause severe pathology, driven by an exaggerated immune response, infection within the intestine generally seems to cause minor or no symptoms. In this review, we compare the disease processes between the lungs and gastrointestinal tract, and what might drive these different responses. As the microbiome is a key part of mucosal barrier sites, we also consider the effect that microbial species may play on infection and the subsequent immune responses. Because of difficulties obtaining tissue samples, there are currently few studies focused on the local mucosal response rather than the systemic response, but understanding the local immune response will become increasingly important for understanding the mechanisms of disease in order to develop better treatments.

Extensive author list of The Oxford-Cardiff COVID-19 Literature Consortium is given in Appendix 1.

## INTRODUCTION

The immune system has been shown to play a critical role in the clearance of severe acute respiratory syndrome coronavirus 2 (SARS-CoV2) and the pathogenesis of resulting coronavirus infectious disease 2019 (COVID-19). Much of the immunological analysis in this disease has focused on the systemic immune response found in the blood. By focusing on recent data from lung and gut, the mucosal sites of infection, we can gain insights into the local immune response, which is likely key to viral clearance,s limiting pathogenesis and vaccine efficacy.

### SARS-CoV2 infection at mucosal sites

SARS-CoV2 enters cells via the angiotensin-converting enzyme (ACE2) [1]. As with other coronaviruses, the spike protein is important for viral attachment and binding to ACE2, and must be cleaved for entry. Transmembrane serine protease 2 has been identified as a cellular serine protease able to cleave the spike protein. The broad spectrum of cell types that express ACE2 goes some way to explaining the wide variety of tissues that can be infected and damaged. ACE2 is particularly abundant on the epithelial cells that line the lung and intestine, specifically secretory goblet cells in the nasal mucosa, Type II pneumocytes in the lung, and absorptive enterocytes in the small intestine [2].

Box 1 Is there a consensus on the topic discussed? And what is the consensus?

There is a broad consensus that an over-exuberant immune response is a key driver of severe disease following SARS-CoV2 infection, and that inducing a moderate immune response will be important in vaccine efficacy. Studies are starting to investigate the immune response at the local site of infection as immunity here will be the first line of defence. There is conflicting data about whether gut involvement in SARS-CoV2 is beneficial or harmful. The microbiome has also become a focus of investigation given the strengthening link between obesity (which is associated with reduced microbial diversity) and worse disease outcome. Mucosal studies remain limited due to the lack of sample access but will become increasingly important in understanding disease.

Box 2 Why does the subject matter?

Understanding the mucosal immune response to SARS-CoV2 will help to enable better treatment of COVID-19 and the development of successful vaccines for prevention. Presently available data on the immunology of COVID-19 generally reflect systemic responses in the blood, with comparatively little on the local immune response within the lung or intestine, both sites of viral infection. Given that intestinal immune responses to infection appear to be regulated, while those in the lung are exaggerated, understanding the similarities and differences between the sites will help to unravel the differing immune responses between mild and severe disease.

In clinical settings, infection with SARS-CoV2 is defined on the basis of viral RNA amplification from nasopharyngeal swab samples. More recently, saliva tests have gained the popularity because of their less invasive nature while maintaining a similar sensitivity [3], due to ACE2 expression in oral cavity epithelial cells [4]. Diagnosis of COVID-19 depends upon a positive test for viral RNA from an oropharyngeal, nasopharyngeal or saliva swab, but it has become clear that the faeces can also contain SARS-CoV2 RNA, in many cases before symptoms appear and long after a patient has tested negative from a conventional swab [5]. Indeed, testing of wastewater for viral RNA is gaining popularity as a way for authorities to quickly identify local hotspots of infection in the community [6].

However, the detection of RNA from SARS-CoV2 in different samples does not necessarily indicate the presence of infectious virus. The gold standard is isolation of virus that can infect an epithelial cell line Vero E *in vitro*, which has been clearly shown in lung samples [7] and documented in faecal samples [8]. High RNA levels may correlate with infectious virus, which has been isolated from nasal swabs, sputum and faeces [7, 9]. However, another study that used small intestine biopsy samples failed to isolate infectious virions [10]. It is also unclear whether any virus from the intestine is infectious once it is egested, or whether it is degraded in the intestine with remaining nucleic acid detectable by qPCR.

**Figure 1:**
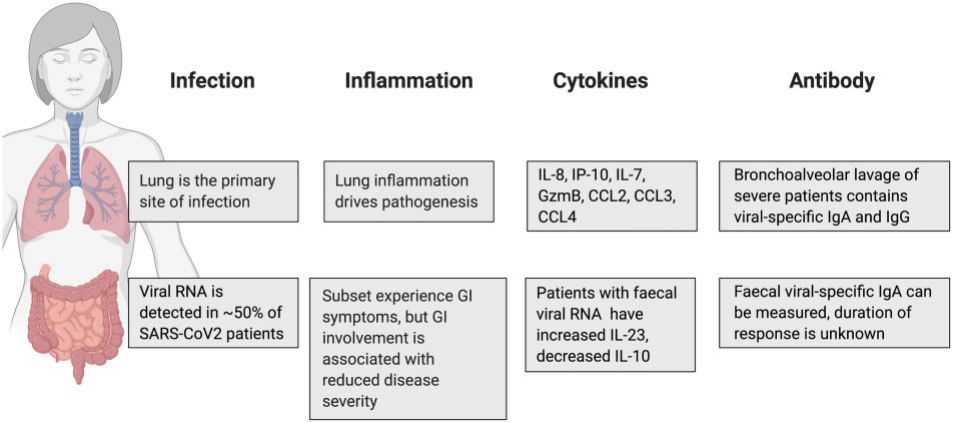
Despite being sites of viral infection with SARS-CoV2, the lungs and gastrointestinal tract show very different responses to the virus. These differences may be important for mounting the most appropriate immune response to the virus

Viral infection of human enterocytes of the small intestine has been shown by microscopy in organoid systems [11] and biopsies [10], indicating that SARS-CoV2 can productively infect cells of the intestine. Around 20% of patients with COVID-19 experience gastrointestinal symptoms, but these are generally mild and include diarrhoea, nausea and vomiting [12]. A COVID-19 symptom tracker has been used to classify six different disease symptom clusters, including a gastrointestinal cluster with diarrhoea but no cough or fever [13]. It remains unclear whether the faecal–oral route is a significant infection risk and whether it may be related to this disease cluster. A summary of the currently understood differences between infection of the lung and gastrointestinal tract is shown in Fig. 1.

### Microbiome and SARS-CoV2

It has long been appreciated that the local microbiota may interact directly with invading viruses or immune cells, or indirectly through metabolite production that modulates the environment [14, 15]. Studies have implicated pharyngeal microbial communities in susceptibility to influenza [16, 17] and secondary bacterial infections [17], but interactions through the gut–lung axis indicate that gut microbes also play a role—the gut bacterium *Lactobacillus paracasei* was able to modulate the immune response to influenza in mice, decreasing damaging inflammatory cell accumulation within the lungs [18]. This all suggests that the microbiome may play a role in modulating both susceptibility and immune responses to viral pathogens. Antibiotic-driven dysbiosis, which would be common among COVID-19 patients, can also affect the immune response to viral infection [19]. In COVID-19, diseases associated with microbial dysbiosis, particularly comorbidities related to metabolic syndrome (including insulin resistance and Type 2 diabetes, obesity and hypertension), have also been implicated in worse outcome. In one study, patients in intensive care units with critical COVID-19 were nearly twice as likely to be obese as the general population [20]. It is therefore unsurprising that many studies are ongoing to investigate associations between lung and gut microbiota and COVID-19. Such studies are complicated by the difficulties of unpicking cause versus effect in an already sick population, as an excessive immune response is likely to affect both local and distal bacterial populations. Initial results indicate decreased microbial diversity in patients with COVID-19 compared with both healthy controls and influenza or pneumonia patients, which may gradually recover over time [21, 22] but can persist long after viral clearance from the respiratory tract [5]. Common commensal microbes such as *Faecalibacterium prausnitzii*, a known producer of short-chain fatty acid metabolites associated with health [23] are significantly transiently reduced in the gut at peak disease, even in patients with mild symptoms [21], and abundance was inversely correlated with lung disease severity and in the absence of gut symptoms [24]. Interestingly, however, a pilot study of the microbiome of infected patients described an increase in *Faecalibacterium* within bronchoalveolar lavage fluid compared with healthy controls [25]. As *Faecalibacterium* is reduced in metabolic disease including obesity and Type 2 diabetes [26], it may be that pre-existing microbial factors alter initial susceptibility to SARS-CoV2 infection, and location of microbes within the body may also play a role. In addition, an increase in certain Clostridial and *Pseudomonas* opportunistic pathogens have been associated with COVID-19 severity, possibly due to increased risk of secondary bacterial infection [21, 22, 24]. Similarly, infection with SARS-CoV2 may also reduce the diversity of the nasopharyngeal microbiome [27], and increase abundance of specific genii that can play a role in mucosal immunity, such as *Prevotella*, the presence of which has been associated with more severe symptoms [28]. However, data are currently limited to small-scale studies, and more prospective, longitudinal analysis of larger cohorts will be required to unpick the many variables that may contribute to microbial changes.

### Disease processes and protection in mucosal tissues

A key feature of severe COVID-19 disease is the extensive lung damage caused by an over-exuberant immune response, but the immune response in the gut remains enigmatic. Despite the evidence for viral infection within the intestine, where a high viral burden might also be expected, gastrointestinal (GI) symptoms are generally mild, although pro-inflammatory interleukin (IL)-23 and IL-8 are increased in faecal samples from COVID-19 patients compared with healthy controls [29]. The presence of diarrhoea in severe patients has been associated with a worse outcome in some studies [13, 30], which could be related either to systemic immune activation or more widespread viral invasion of tissues. Interestingly, another study using three patient cohorts found an association between GI symptoms and reduced disease severity and mortality even when comorbidities were accounted for [10]. A few patients with GI symptoms who underwent endoscopic evaluation had no evidence of mucosal inflammation despite the epithelial infection. GI infection was associated with reduced levels of circulating pro-inflammatory cytokines including IL-1β, tumour necrosis factor (TNF)α and IL-6, but increased IL-7, a critical T-cell development and survival cytokine [31]. This would suggest that GI infection may lead to a less inflammatory response than in the lungs in adults. However, in multisystem inflammatory system in children, a rare but severe disease following SARS-CoV2 infection in children, a common feature is gastrointestinal symptoms rather than lung involvement [32]. Symptoms seem to occur up to 4 weeks after COVID-19 exposure so are likely to involve an aberrant immune response rather than direct damage from the virus [33]. Treatment with intravenous immunoglobulin (Ig), anti-IL-6 or anti-IL-1 has had beneficial effects [34], suggesting that in these children there is an excessive immune response despite the gastrointestinal involvement.

Neutrophils have emerged as potentially important regulators of lung disease, due to the significant formation of neutrophil extracellular traps (NETs) in COVID-19 patients in respiratory failure, and the correlation between neutrophilia and poor outcome [35]. NETs trap viral particles upon neutrophil death by NETosis [36], but their extrusion can also cause coagulation, a feature of severe COVID-19. An increase in both neutrophil and pro-inflammatory macrophage accumulation in bronchoalveolar fluid has been noted in severe COVID-19 patients [37] along with an increase in neutrophil degranulation [38]. Post-mortem examination of lung samples from patients who died with respiratory involvement showed a significant macrophage and monocyte exudate in alveolar cavities, as well as some neutrophil and lymphocyte accumulation [39–42].

The cytokine environment induced by the different immune cell players is also likely to play a critical role in outcome. Treatment with the steroid Dexamethasone demonstrated that inhibiting the immune response can improve survival in severely ill patients [43]. Damage to alveolar epithelial cells is likely to stimulate early responding monocytes, macrophages and neutrophils to make high levels of IL-1b, IL-6, IL-18, interferon (IFN)γ and TNFα, which can act downstream on T-cell activation and differentiation. The presence of high serum IL-6 has been associated with poor outcome [44], but treatment with anti-IL-6, however, has shown mixed results [45] and may be beneficial only in a subset of patients with the highest hyperinflammation. Type I interferons are directly induced by viral infection and are the important component of anti-viral responses. About 15% of severe cases have been associated with genetic variants in the interferon pathway or auto-antibodies against the cytokines [46, 47]. Circulating IFNβ expression has been shown to be reduced in all patients with COVID-19 regardless of disease severity [48]. As Type I IFNs are potent stimulators of natural killer and T cells, perhaps it is unsurprising that overall there appears to be a decrease in natural killer cells and all T cells in COVID-19 lungs. Most notably CD8^+^ T cells are reduced in the lung and bronchoalveolar fluid in severe COVID-19, as well as in the blood [48], and these cells showed fewer markers of proliferation and tissue residency than those found in moderate or no disease [37]. Indeed, a broad T-cell memory of the virus is associated with a milder course of disease [49] and local lung CD4 T-cell responses are associated with survival from severe disease [41]. This could suggest that in severe disease there are fewer SARS-CoV2-specific anti-viral CD8^+^ T cells within the tissues but continued innate cell activation from high viral load. Treatment with IFNβ subcutaneously may shorten disease duration and reduce symptoms [50], but inhalation directly into the lungs may be a better method of delivery, as this is where the biggest problem lies. Indeed, Synairgen has reported (in a pre-print) that treatment with inhaled IFNβ reduced the development of severe disease by up to 79% in an early trial [51]. It may be that mucosal administration by inhalation of other treatments leads to a better response than when given systemically.

Although lung T cells decrease in severe COVID-19, there appears to be an increase in B cells [48], reflecting their importance in antibody production. A number of studies have measured immunoglobulin levels in the serum, particularly IgG and IgA, both of which are found in convalescent patients. However, as IgG comprises the majority of serum immunoglobulin, current antibody tests use IgM and IgG only, although IgA is mainly found in mucosal tissues such as the lung, and may therefore be of as great or greater importance. Limited data hint at early neutralizing IgA responses being associated with milder infection [52, 53], and that IgA peaks earlier than IgG [53]. Analysis of faecal samples from COVID-19 patients suggests viral-specific antibody responses can develop in the gut [29], but the effect of this antibody on systemic levels is unknown. Serum antibody appears to wane in the weeks and months post-infection [54], however, it is unclear what happens within the lung tissue. Antibody concentrations in the saliva suggest IgA rapidly wanes, but IgG is maintained, at least in the oral cavity [55]. Measurement of SARS-CoV2-specific antibody and memory B and T cells within mucosal tissues rather than just serum may give a more accurate picture as to the impact of antibody on recovery and protection from reinfection. Given the difficulties in obtaining mucosal tissue samples from humans with COVID-19, and the advent of a host of animal models including ferrets, non-human primates and the use of a mouse-adapted virus [56], investigation of mucosal responses in animal models may help to increase our understanding of the immune response to SARS-CoV2 in lung and intestinal sites.

Antibody responses are critical for design and interpretation of the efficacy of vaccine trials, as they are one of the primary readouts for an immune response, and length of antibody response is an important consideration for vaccine success. However, a successful vaccine is likely to have wider effects than the antibody induction alone, possibly also inducing a memory T-cell response that can contribute to protection. Early results of ChAdOx1 nCoV-19 vaccination in rhesus macaques indicated that while the animals were protected from pneumonia, nasal carriage of the virus remained at the same level as non-vaccinated animals [21], hence, animals may be protected from the worst COVID-19 symptoms but remain infectious to others. A single dose of ChAd vaccine can lead to a local sterilizing response [57] when given intranasally rather than intramuscularly, highlighting the importance of induction of a local mucosal immune response.

## CONCLUSION

Although the great strides have been made in our understanding of SARS-CoV2 infection and subsequent disease in the past 9 months, the study of mucosal immunity will be critical to future endeavours to prevent and treat disease.

## DATA AVAILABILITY STATEMENT

No new data were generated or analysed in support of this review.

## APPENDIX 1: THE OXFORD-CARDIFF COVID-19 LITERATURE CONSORTIUM—EXTENSIVE AUTHOR LIST

David J. Ahern^1^, Hannah Almuttaqi^1^, Dominic S. Alonzi^2^, Aljawharah Alrubayyi^3^, Ghada Alsaleh^1^, Valentina M. T. Bart^4^, Vicky Batchelor^1^, Rebecca Bayliss^5^, Dorothée L. Berthold^1^, Jelena S. Bezbradica^1^, Tehmina Bharuchq^2^, Helene Borrmann^3^, Mariana Borsa^1^, Rowie Borst^1^, Juliane Brun^2^, Stephanie Burnell^4^, Lorenzo Capitani^4^, Athena Cavounidis^6^, Lucy Chapman^4^, Anne Chauveau^1^, Liliana Cifuentes^1^, Amy Susan Codd^4^, Ewoud Bernardus Compeer^1^, Clarissa Coveney^1^, Amy Cross^7^, Sara Danielli^1^, Luke C. Davies^4^, Calliope A. Dendrou^8^, Sandra Dimonte^4^, Ruban Rex Peter Durairaj^4^, Lynn B. Dustin^1^, Arthur Dyer^9^, Ceri Fielding^4^, Fabian Fischer^1^, Awen Gallimore^4^, Sarah Galloway^4^, Anís Gammage^1^, Ester Gea-Mallorquí^1^, Andrew Godkin^4^, Stephanie Jean Hanna^4^, Cornelia Heuberger^1^, Sarah Hulin-Curtis^4^, Fadi Issa^7^, Emma Jones^4^, Ruth Jones^10^, Kristin Ladell^4^, Sarah N. Lauder^4^, Kate Liddiard^5^, Petros Ligoxygakis^11^, Fangfang Lu^12^, Bruce MacLachlan^4^, Shayda Maleki-Toyserkani^4^, Elizabeth H. Mann^1^, Anna M. Marzeda^1^, Reginald James Matthews^13^, Julie M. Mazet^1^, Anita Milicic^14^, Emma Mitchell^4^, Owen Moon^4^, Van Dien Nguyen^4^, Miriam O'Hanlon^1^, Clara Eléonore Pavillet^15^, Dimitra Peppa^3^, Ana Pires^4^, Eleanor Pring^4^, Max Quastel^16^, Sophie Reed^4^, Jan Rehwinkel^17^, Niamh Richmond^1^, Felix Clemens Richter^1^, Alice J. B. Robinson^1^, Patrícia R. S. Rodrigues^4^, Pragati Sabberwal^4^, Arvind Sami^18^, Raphael Sanches Peres^1^, Quentin Sattentau^12^, Barbora Schonfeldova^1^, David Oliver Scourfield^4^, Tharini A. Selvakumar^16^, Freya R. Shepherd^4^, Cariad Shorten^15^, Anna Katharina Simon^1^, Adrian L. Smith^19^, Alicia Teijeira Crespo^5^, Michael Tellier^12^, Emily Thornton^17^, Lion F. K. Uhl^1^, Erinke van Grinsven^1^, Angus K. T. Wann^1^, Richard Williams^1^, Joseph D. Wilson^15^, Dingxi Zhou^1^ and Zihan Zhu^12^

### Affiliations of Consortium members

^1^Kennedy Institute of Rheumatology, Nuffield Department of Orthopaedics, Rheumatology and Musculoskeletal Sciences, University of Oxford, Oxford, UK, ^2^Oxford Glycobiology Institute, Department of Biochemistry, University of Oxford, Oxford, UK, ^3^Nuffield Department of Clinical Medicine, Nuffield Department of Medicine, University of Oxford, Oxford, UK, ^4^Division of Infection and Immunity, School of Medicine, Cardiff University, Cardiff, UK, ^5^Division of Cancer and Genetics, School of Medicine, Cardiff University, UK, ^6^Translational Gastroenterology Unit, Nuffield Department of Medicine, University of Oxford, Oxford, UK, ^7^Nuffield Department of Surgical Sciences, University of Oxford, Oxford, UK, ^8^Wellcome Centre for Human Genetics, Nuffield Department of Medicine, University of Oxford, Oxford, UK, ^9^Department of Oncology, University of Oxford, Oxford, UK, ^10^Dementia Research Institute, Haydn Ellis Building, Cardiff University, Cardiff, UK, ^11^Department of Biochemistry, University of Oxford, Oxford, UK, ^12^Sir William Dunn School of Pathology, Medical Science Division, University of Oxford, Oxford, UK, ^13^Centre for Medical Education, School of Medicine, Cardiff University, Cardiff, UK, ^14^The Jenner Institute, Nuffield Department of Medicine, University of Oxford, Oxford, UK, ^15^Medical Sciences Division, University of Oxford, Oxford, UK, ^16^Nuffield Department of Medicine, University of Oxford, Oxford, UK, ^17^Medical Research Council Human Immunology Unit, Medical Research Council Weatherall Institute of Molecular Medicine, Radcliffe Department of Medicine, University of Oxford, Oxford, UK, ^18^Botnar Research Centre, Nuffield Department of Orthopaedics, Rheumatology and Musculoskeletal Sciences, University of Oxford, Oxford, UK and ^19^Department of Zoology, University of Oxford, UK

## References

[1] Hoffmann M , Kleine-Weber H, Schroeder S et al SARS-CoV-2 cell entry depends on ACE2 and TMPRSS2 and is blocked by a clinically proven protease inhibitor. Cell 2020, doi:10.1016/j.cell.2020.02.052. 10.1016/j.cell.2020.02.052 PMC7102627 32142651

[2] Ziegler CGK , Allon SJ, Nyquist SK et al SARS-CoV-2 receptor ACE2 is an interferon-stimulated gene in human airway epithelial cells and is detected in specific cell subsets across tissues. Cell 2020. doi:10.1016/j.cell.2020.04.035. 10.1016/j.cell.2020.04.035 PMC7252096 32413319

[3] Wang H , Liu Q, J Hu et al Nasopharyngeal swabs are more sensitive than oropharyngeal swabs for COVID-19 diagnosis and monitoring the SARS-CoV-2 load. Front Med 2020. doi:10.3389/fmed.2020.00334. 10.3389/fmed.2020.00334 PMC7314917 32626720

[4] Braz-Silva PH , Pallos D, Giannecchini S et al SARS-CoV-2: what can saliva tell us? Oral Dis 2020. doi:10.1111/odi.13365. 10.1111/odi.13365 PMC7264628 32311181

[5] Wu Y , Guo C, Tang L et al Prolonged presence of SARS-CoV-2 viral RNA in faecal samples. Lancet Gastroenterol Hepatol 2020;5:434–5. 32199469 10.1016/S2468-1253(20)30083-2 PMC7158584

[6] Peccia J , Zulli A, Brackney DE et al SARS-CoV-2 RNA concentrations in primary municipal sewage sludge as a leading indicator of COVID-19 outbreak dynamics. medRxiv 2020. doi:10.1101/2020.05.19.20105999.

[7] Wölfel R , Corman VM, Guggemos W et al Virological assessment of hospitalized patients with COVID-2019. Nature 2020;581:465–9. 32235945 10.1038/s41586-020-2196-x

[8] Zhang Y , Chen C, Zhu S et al Isolation of 2019-nCoV from a stool specimen of a laboratory-confirmed case of the coronavirus disease 2019 (COVID-19). China CDC Wkly 2020;2:123–4. PMC8392928 34594837

[9] Xiao F , Sun J, Xu Y et al Infectious SARS-CoV-2 in feces of patient with severe COVID-19. Emerg Infect Dis 2020;26:1920–2. 32421494 10.3201/eid2608.200681 PMC7392466

[10] Livanos AE , Jha D, Cossarini F et al Gastrointestinal involvement attenuates COVID-19 severity and mortality. medRxiv 2020. doi:10.1101/2020.09.07.20187666.

[11] Lamers MM , Beumer J, Vaart J et al SARS-CoV-2 productively infects human gut enterocytes. Science 2020. doi:10.1126/science.abc1669. 10.1126/science.abc1669 PMC7199907 32358202

[12] Docherty AB, Harrison EM, Green CA *et al.* Features of 20 133 UK patients in hospital with covid-19 using the ISARIC WHO Clinical Characterisation Protocol: Prospective observational cohort study. *BMJ* 2020;369, DOI: 10.1136/bmj.m1985. 10.1136/bmj.m1985 PMC7243036 32444460

[13] Sudre CH , Lee K, Lochlainn MN et al Symptom clusters in Covid19: a potential clinical prediction tool from the COVID symptom study app. MedRxiv 2020. 10.1126/sciadv.abd4177 PMC7978420 33741586

[14] Rooks MG , Garrett WS. Gut microbiota, metabolites and host immunity. Nat Rev Immunol 2016;16:341–52. 27231050 10.1038/nri.2016.42 PMC5541232

[15] Domínguez-Díaz C , García-Orozco A, Riera-Leal A et al Microbiota and its role on viral evasion: is it with us or against us? Front Cell Infect Microbiol 2019;9:256. 31380299 10.3389/fcimb.2019.00256 PMC6657001

[16] Lee KH , Gordon A, Shedden K et al The respiratory microbiome and susceptibility to influenza virus infection. PLoS One 2019. doi:10.1371/journal.pone.0207898. 10.1371/journal.pone.0207898 PMC6326417 30625134

[17] Mulcahy ME , McLoughlin RM. Staphylococcus aureus and influenza a virus: partners in coinfection. MBio 2016;7. doi:10.1128/mBio.02068-16. 10.1128/mBio.02068-16 PMC5156308 27965455

[18] Belkacem N , Serafini N, Wheeler R et al Lactobacillus paracasei feeding improves immune control of influenza infection in mice. PLoS One 2017;12:e0184976. 28931041 10.1371/journal.pone.0184976 PMC5607164

[19] Grayson MH , Camarda LE, Hussain SRA et al Intestinal microbiota disruption reduces regulatory T cells and increases respiratory viral infection mortality through increased IFNγ production. Front Immunol 2018;9:1587. 30042764 10.3389/fimmu.2018.01587 PMC6048222

[20] Caussy C , Pattou F, Wallet F et al Prevalence of obesity among adult inpatients with COVID-19 in France. Lancet Diabetes Endocrinol 2020;8:562–4. 32437642 10.1016/S2213-8587(20)30160-1 PMC7234780

[21] Tao W , Zhang G, Wang X et al Analysis of the intestinal microbiota in COVID-19 patients and its correlation with the inflammatory factor IL-18 and SARS-CoV-2-specific IgA. medRxiv 2020. 10.1016/j.medmic.2020.100023 PMC7832617 34173452

[22] Xu R , Lu R, Zhang T et al Temporal dynamics of human respiratory and gut microbiomes during the course of COVID. medRxiv 2020. doi: 10.1101/2020.07.21.20158758

[23] Duncan SH , Hold GL, Harmsen HJM et al Growth requirements and fermentation products of Fusobacterium prausnitzii, and a proposal to reclassify it as *Faecalibacterium prausnitzii* gen. nov., comb. nov. Int J Syst Evol Microbiol 2002;52:2141–6. 12508881 10.1099/00207713-52-6-2141

[24] Zuo T , Zhang F, Lui GCY et al Alterations in gut microbiota of patients with COVID-19 during time of hospitalization. Gastroenterology 2020;159:944–955.e8. 32442562 10.1053/j.gastro.2020.05.048 PMC7237927

[25] Han Y , Jia Z, Shi J et al The active lung microbiota landscape of COVID-19 patients. medRxiv 2020. doi:10.1101/2020.08.20.20144014. 10.34172/bi.2021.23378 PMC8905590 35411293

[26] Geva-Zatorsky N , Sefik E, Kua L et al Mining the human gut microbiota for immunomodulatory organisms. Cell 2017;168:928–943.e11. 28215708 10.1016/j.cell.2017.01.022 PMC7774263

[27] Mostafa HH , Fissel JA, Fanelli B et al Metagenomic next-generation sequencing of nasopharyngeal specimens collected from confirmed and suspect COVID-19 patients. MBio 2020. doi:10.1128/mBio.01969-20. 10.1128/mBio.01969-20 PMC7686804 33219095

[28] Paz Ventero M , Ricardo Castro Cuadrat R, Vidal I et al Nasopharyngeal microbial communities of patients infected with SARS-COV-2 that 1 developed COVID-19. bioRxiv 2020. doi:10.1101/2020.12.01.407486. 10.3389/fmicb.2021.637430 PMC8010661 33815323

[29] Britton GJ , Chen-Liaw A, Cossarini F et al SARS-CoV-2-specific IgA and limited inflammatory cytokines are present in the stool of select patients with acute COVID-19. medRxiv 2020.

[30] Pan L , Mu M, Yang P et al Clinical characteristics of COVID-19 patients with digestive symptoms in Hubei, China: A descriptive, cross-sectional, multicenter study. Am J Gastroenterol 2020. doi:10.14309/ajg.0000000000000620. 10.14309/ajg.0000000000000620 PMC7172492 32287140

[31] Barata JT , Durum SK, Seddon B. Flip the coin: IL-7 and IL-7R in health and disease. Nat Immunol 2019;20:1584–93. 31745336 10.1038/s41590-019-0479-x

[32] Feldstein LR , Rose EB, Horwitz SM et al Multisystem inflammatory syndrome in U.S. children and adolescents. N Engl J Med 2020;383:334–46. 32598831 10.1056/NEJMoa2021680 PMC7346765

[33] Jiang L , Tang K, Levin M et al COVID-19 and multisystem inflammatory syndrome in children and adolescents. Lancet Infect Dis 2020. doi:10.1016/S1473-3099(20)30651-4. 10.1016/S1473-3099(20)30651-4 PMC7431129 32818434

[34] Hennon TR , Penque MD, Abdul-Aziz R et al COVID-19 associated Multisystem Inflammatory Syndrome in Children (MIS-C) guidelines; a Western New York approach. Prog Pediatr Cardiol 2020. doi:10.1016/j.ppedcard.2020.101232. 10.1016/j.ppedcard.2020.101232 PMC7244417 32837142

[35] Wang D , Hu B, Hu C et al Clinical characteristics of 138 hospitalized patients with 2019 novel coronavirus-infected pneumonia in Wuhan, China. J Am Med Assoc 2020. doi:10.1001/jama.2020.1585. 10.1001/jama.2020.1585 PMC7042881 32031570

[36] Brinkmann V , Reichard U, Goosmann C et al Neutrophil extracellular traps kill bacteria. Science 2004. doi:10.1126/science.1092385. 10.1126/science.1092385 15001782

[37] Liao M , Liu Y, Yuan J et al Single-cell landscape of bronchoalveolar immune cells in patients with COVID-19. Nat Med 2020. doi:10.1038/s41591-020-0901-9. 10.1038/s41591-020-0901-9 32398875

[38] Rosa BA , Ahmed M, Singh DK et al IFN signaling and neutrophil degranulation transcriptional signatures are induced during SARS-CoV-2 infection. bioRxiv 2020. 10.1038/s42003-021-01829-4 PMC7935909 33674719

[39] Wang C , Xie J, Zhao L et al Alveolar macrophage dysfunction and cytokine storm in the pathogenesis of two severe COVID-19 patients. EBioMedicine 2020. doi:10.1016/j.ebiom.2020.102833. 10.1016/j.ebiom.2020.102833 PMC7305897 32574956

[40] Fox SE , Akmatbekov A, Harbert JL et al Pulmonary and cardiac pathology in African American patients with COVID-19: an autopsy series from New Orleans. Lancet Respir Med 2020. doi:10.1016/S2213-2600(20)30243-5. 10.1016/S2213-2600(20)30243-5 PMC7255143 32473124

[41] Szabo PA , Dogra P, Gray JI et al Analysis of respiratory and systemic immune responses in COVID-19 reveals mechanisms of disease pathogenesis. medRxiv 2020. doi:1003:2020.10.15.20208041.

[42] Figueiredo Rendeiro A , Ravichandran H, Bram Y et al The spatio-temporal landscape of lung pathology in SARS-CoV-2 infection. medRxiv 2020. DOI: 10.1101/2020.10.26.20219584.

[43] RECOVERY Collaborative Group; Peter Horby, Wei Shen Lim, Jonathan R Emberson, Marion Mafham, Jennifer L Bell, Louise Linsell, Natalie Staplin, Christopher Brightling, Andrew Ustianowski, Einas Elmahi, Benjamin Prudon, Christopher Green, Timothy Felton, David Chadwick, Kanchan Rege, Christopher Fegan, Lucy C Chappell, Saul N Faust, Thomas Jaki, Katie Jeffery, Alan Montgomery, Kathryn Rowan, Edmund Juszczak, J Kenneth Baillie, Richard Haynes, Martin J Landray. Dexamethasone in hospitalized patients with Covid-19—preliminary report. N Engl J Med 2020. doi:10.1056/nejmoa2021436.

[44] Wu C , Chen X, Cai Y et al Risk factors associated with acute respiratory distress syndrome and death in patients with coronavirus disease 2019 pneumonia in Wuhan, China. JAMA Intern Med 2020. doi:10.1001/jamainternmed.2020.0994. 10.1001/jamainternmed.2020.0994 PMC7070509 32167524

[45] Campochiaro C , Dagna L. The conundrum of interleukin-6 blockade in COVID-19. Lancet Rheumatol 2020. doi:10.1016/S2665-9913(20)30287-3. 10.1016/S2665-9913(20)30287-3 PMC7428300 32838322

[46] Bastard P , Rosen LB, Zhang Q et al Auto-antibodies against type I IFNs in patients with life-threatening COVID-19. Science 2020. doi:10.1126/science.abd4585. 10.1126/science.abd4585 PMC7857397 32972996

[47] Zhang Q , Bastard P, Liu Z et al Inborn errors of type I IFN immunity in patients with life-threatening COVID-19. Science 2020. doi:10.1126/science.abd4570. 10.1126/science.abd4570 PMC7857407 32972995

[48] Hadjadj J , Yatim N, Barnabei L et al Impaired type I interferon activity and inflammatory responses in severe COVID-19 patients. Science 2020. doi:10.1126/science.abc6027. 10.1126/science.abc6027 PMC7402632 32661059

[49] Peng Y , Mentzer AJ, Liu G et al Broad and strong memory CD4+ and CD8+ T cells induced by SARS-CoV-2 in UK convalescent individuals following COVID-19. Nat Immunol 2020. doi:10.1038/s41590-020-0782-6. 10.1038/s41590-020-0782-6 PMC7611020 32887977

[50] Hung IFN , Lung KC, Tso EYK et al Triple combination of interferon beta-1b, lopinavir–ritonavir, and ribavirin in the treatment of patients admitted to hospital with COVID-19: an open-label, randomised, phase 2 trial. Lancet 2020. doi:10.1016/S0140-6736(20)31042-4. 10.1016/S0140-6736(20)31042-4 PMC7211500 32401715

[51] Monk PD, Marsden RJ, Tear VJ *et al.* Safety and efficacy of inhaled nebulised interferon beta-1a (SNG001) for treatment of SARS-CoV-2 infection: a randomised, double-blind, placebo-controlled, phase 2 trial. *Lancet Respir Med* 2020, DOI: 10.1016/S2213-2600(20)30511-7. 10.1016/S2213-2600(20)30511-7 PMC7836724 33189161

[52] Sterlin D , Mathian A, Miyara M et al IgA dominates the early neutralizing antibody response to SARS-CoV-2. medRxiv 2020. 10.1126/scitranslmed.abd2223 PMC7857408 33288662

[53] Ma H , Zeng W, He H et al COVID-19 diagnosis and study of serum SARS-CoV-2 specific IgA, IgM and IgG by a quantitative and sensitive immunoassay. medRxiv 2020.

[54] Long QX , Tang XJ, Shi QL et al Clinical and immunological assessment of asymptomatic SARS-CoV-2 infections. Nat Med 2020. doi:10.1038/s41591-020-0965-6. 10.1038/s41591-020-0965-6 32555424

[55] Isho B, Abe KT, Zuo M *et al.* Persistence of serum and saliva antibody responses to SARS-CoV-2 spike antigens in COVID-19 patients. *Sci Immunol* 2020;5, DOI: 10.1126/sciimmunol.abe5511. 10.1126/sciimmunol.abe5511 PMC8050884 33033173

[56] Muñoz-Fontela C , Dowling WE, Funnell SGP et al Animal models for COVID-19. Nature 2020. doi:10.1038/s41586-020-2787-6. 10.1038/s41586-020-2787-6 PMC8136862 32967005

[57] Hassan AO , Kafai NM, Dmitriev IP et al A single-dose intranasal ChAd vaccine protects upper and lower respiratory tracts against SARS-CoV-2. Cell 2020. doi:10.1016/j.cell.2020.08.026. 10.1016/j.cell.2020.08.026 PMC7437481 32931734

